# Reports of Meetings

**Published:** 1952-10

**Authors:** 


### *Reports of Meetings*

### BRISTOL MEDICO-CHIRURGICAL SOCIETY

*At the Royal Fort on January 14th,* 1953

### Aims and Limitations of Insulin Therapy

**Dr. Wilfrid Oakley** (King's College Hospital) began by considering, in the light of recent researches, the subdivision of diabetics into different clinical types; in the same connection reference was made to the work of Bornstein and Lawrence on the insulin content of the plasma in diabetes. The speaker next defined the aims of treatment by insulin and stressed the importance of estimating the blood sugar and testing the urine at times when insulin action was at its maximum.

The various arrangements and combinations of insulins available were briefly reviewed, and the type of patient for which each was most suitable was discussed. Special emphasis was laid on the advantages in difficult cases, such as children and pregnant diabetics, of the use of a single injection of soluble insulin before breakfast and a mixture of soluble and protamine zinc insulin before tea or the evening meal; the dose of protamine zinc insulin should be as small as possible, the aim being to keep the early morning specimen of urine free of ketone bodies. The speaker went on to consider the incidence of retinitis, albuminuria, hypertension and Kimmelstiel Wilson syndrome and showed from a large series of young diabetics that these complications are related to the duration and not to the severity of the diabetes. The high proportion of such cases affected showed that the present methods of treatment are far from perfect, but recent American work seems to confirm the clinical impression that good control of the diabetes lowers the incidence of these complications.

In dealing with the treatment of severe ketosis and diabetic coma, Dr. Oakley warned his audience against the danger of giving very large and frequently repeated doses of insulin without adequate blood sugar control; he considered this recently popular practice both unnecessary and dangerous. The importance of due attention to fluid and electrolyte balance was stressed, and the indications for the use of potassium in the treatment of coma explained.

The local reactions to insulin injections were illustrated by a series of photographs showing severe bilateral symmetrical insulin atrophy, fibro-fatty insulin tumours and the effects of intracutaneous injections.

In conclusion, a brief account was given of a recent therapeutic trial of the new Danish insulin produced by Novo Laboratories in Copenhagen as a result of the researches of Hallas Möller and his co-workers. Three new preparations of increasing duration and decreasing intensity of action had been produced under the names Semi-Lente, Lente and Ultra-Lente, the preparation tried at King's College Hospital being the intermediate Lente insulin. Graphs of blood sugars taken at 8 a.m., noon, 6 p.m., and 9 p.m. were shown from which it was evident that this new insulin not only has a prolonged action over twenty-four hours or more, but also acts with sufficient rapidity to control the noon blood sugar level. It seems from the small series of cases so far treated with Lente insulin that this preparation possesses all the advantages of a mixed dose of soluble and protamine zinc insulin with the additional virtue of a more regular and dependable action.

### DEVON AND EXETER MEDICO-CHIRURGICAL SOCIETY

### Clinico-Pathological Demonstration

A clinico-pathological demonstration was held on Thursday, November 20th, 1952, at which the following cases were shown:

1. **Adrenal Tumour:** Dr. G. M. Colson, Dr. G. Stewart Smith.

2. **Mixed Growth of Uterus:** Mr. D. Jefferiss, Dr. G. Stewart Smith.
3. **Rupture of Oesophagus:** Mr. J. M. Large, Dr. G. Stewart Smith.
4. **Talc Granulomata:** Mr. P. M. G. Russell, Dr. G. Stewart Smith.
5. **Mere Monstrosities—Single and Double:** Dr. G. Stewart Smith.

### Common Out-Patient Problems

**Dr. W. A. Lister** spoke of common out-patient problems, at a meeting on December 18th, 1952. The first was that of the child who persistently coughs at night. This cough starts at about the age of two years, often after pertussis and pneumonia. The patient is quick, alert, impatient, growing fast, rather pale, and intelligent. There are complaints of abdominal pains and persistent nasal catarrh. Enquiry reveals that the mother has taken the child to many doctors, hospitals and clinics, but still the cough continues. These are the main causative factors: an infective focus in the throat, nasopharynx or sinuses; a psycho-somatic factor, *e.g.* the patient is often the only child of anxious parents; a dietetic factor, usually an intolerance of fat. If these patients are recognized as early asthmatics other things fall into place. Focal infection must be dealt with: this will improve but not cure the patient. The psychological side should receive attention, perhaps at a child guidance clinic. Dietetic measures consist in reducing fat and increasing carbohydrate intake. Skin tests are disappointing, but the common allergens such as feathers, hair and pollen should be avoided as much as possible. Thus, the removal of the domestic cat sometimes produces striking improvement. The breathing exercises advocated in the booklet of the Asthma Research Council are of great help. Dr. Lister's second problem was that of the mild melancholic. Such patients often complained of vertical headache or pressure, or of a boring pain in the temporal region. The condition is apt to follow the receipt of bad news and to occur in elderly prostatics. A careful history will often elicit evidence of previous attacks. Skin rashes, night waking in the small hours, and withdrawal of interest from customary occupations are characteristic. Organic disease must, of course, be carefully excluded before a diagnosis of melancholia is made. Treatment consists in attacking the night waking with phenobarbitone and countering the consequent morning hangover with benzedrine. (The depression is worse in the morning.) If these measures fail E.C.T. often succeeds.

Dr. Lister's third problem was that of back strain producing persistent and otherwise inexplicable pain. Such pain may be due to minor degrees of root irritation on the concave side of a scoliosis. Poker back follows prolonged and unnecessary decubitus. Both types respond well to physiotherapy.

In proposing a vote of thanks Dr. Charles Wroth deplored the overlapping of radiological services as a result of which the same patient might be repeatedly and unnecessarily X-rayed in various clinics and hospitals (or even in the same hospital) owing to lack of adequate liaison.

### SOUTH WEST ORTHOPAEDIC CLUB

The South West Orthopaedic Club met at Winford Orthopaedic Hospital, Bristol, on Saturday, November 22nd, under the Chairmanship of Mr. A. Eyre Brook. In the morning the forty-five members were taken on Ward Rounds by the staff of the hospital. Many cases were demonstrated, among which was the reduction of congenital dislocation of the hip by traction and abduction in flexion on a gallows splint, and arthroplasty of the knee for osteoarthritis using nylon sheets. In the operating theatre an arthroplasty of the hip using an acrylic prosthesis was also demonstrated.

**Mr. K. H. Pridie** reported a new method of arthrodesis of the ankle using a $1\frac{1}{2}$ in. auger bit. The joint was approached through a median incision; the internal malleolus was removed with an osteotome. A cavity was then reamed out in the lower end of the tibia and upper surface of the astragalus. The cavity was filled with bone chips from the tibia, or if the bone was very sclerotic, with iliac bone chips. Sometimes the remains of the internal malleolus were inserted as a key graft. It was possible to correct valgus or varus deformity easily as the ankle was " plastic ". The operation had been done twelve times for various conditions, but never for tuberculosis. Union was sound in about three months and there had been no failures.

**Mr. M. P. McCormack** reviewed the correlation of the neurological signs and operative findings in fifty cases of prolapse of lumbar intervertebral discs. All the cases had been subject to the usual neurological examination before operation.

There had been forty-two positive findings of disc lesions.

In one-third of cases the neurological signs were unhelpful or wrong in indicating level. Two-thirds of the thirty-two cases, where the lesion was situated at the fifth space, had a diminished or absent ankle jerk and half of this group had sensory loss in the first sacral dermatome distribution. The findings in association with the 4/5 disc were not so helpful. In the discussion that followed, Mr. Mervyn Evans spoke of the difficulty of getting miners who had disc lesions back to work and said that this appeared to be directly related to the form in which compensation was paid, those with weekly payments rarely returning, whilst many of those with lump sum payments did so.

**Mr. Keith Lucas** demonstrated a case of chronic arthritis of the hip joint which followed changes in the hands and ankles suggestive of rheumatoid arthritis. The patient had been treated with gold, streptomycin and P.A.S. The course of the hip disease shown by serial X-ray examination strongly suggested a tuberculous infection, but a synovial biopsy had not been done. He considered the other joint changes to be an allergic manifestation of a tuberculous hip infection, although rheumatoid arthritis of the hip sometimes resembled tuberculosis.

**Mr. Durbin** emphasized the importance of proving the diagnosis before any patient was labelled tuberculous as there was a stigma attached to the disease and no person could be accepted by the services or for life assurance purposes with a past history of this trouble. He urged therefore that synovial biopsy should be carried out if other means of diagnosis had failed. Other speakers mentioned the value of gland biopsy.

**Mr. Fowler** reported three cases of fracture of the head of the radius where there had virtually been a traumatic excision of the head of the radius, the fragments being exploded through the capsule into the extra-articular tissues. In such cases it was unnecessary to carry out surgical removal of the fragments, and indeed it was meddlesome, as a good result would ensue by conservatism. All these cases had about three-quarters range of flexion and extension and full rotation.

After tea a discussion was held on the complications of the treatment of club foot, which was opened by Mr. K. H. Pridie and Mr. Bastow.

**Mr. Pridie** stated that he felt that all club feet were amenable to treatment, and that failure was the fault of the surgeon. Treatment might be too active.

**Mr. Bastow** also emphasized that the complications are essentially those of treatment. During the first six weeks manipulations should be little and often, extreme gentleness being used. The soft tissues were frequently damaged by forcible manipulation—the skin was split, pressure sores were produced and Volkmann's ischaemic contracture could develop. He mentioned that Kite's plasters could sometimes produce severe circulatory trouble. Overcorrection should only be obtained at the end of three months. X-rays were shown demonstrating the bad effect of forcible manipulations over a long period on the growth of the lower tibial epiphysis and astragalus. Considerable interest was shown, but owing to limited time, the discussion had to be curtailed.

### SOUTH WEST PAEDIATRIC CLUB

A week-end meeting was held at the Royal United and Manor Hospitals, Bath, on November 22nd and 23rd.

The Paediatric Unit and other departments were visited before the clinical meeting, at which cases presented by Drs. J. Apley, L. H. Hoffmann, E. W. Miles, H. R. E. Wallis and Mr. P. Sames, and X-ray demonstrations by Drs. H. J. Johnson and A. C. P. D. Thomson, were discussed.

**Mr. J. Bastow** gave a paper on *Acute Wry-neck in Childhood.* He reviewed the aetiological factors of this not uncommon condition, which may present as a paediatric rather than an orthopaedic problem, and in which the diagnostic features are mainly clinical. Excellent illustrative case-histories were presented, and the safe and efficient method of treatment by neck traction was clearly demonstrated on some very co-operative patients.

**Dr. H. R. E. Wallis,** under the title, *The Chinless Child,* gave the results of his investigation into all forms of mal-occlusion. What has been regarded as an exclusively dental problem has, in fact, many medical applications. As the processes of natural selection are countered by the technique of modern civilization, the defect may become increasingly prevalent. The commonest underlying factors in the production of the defect were found to be heredity, thumb or finger sucking, and prematurity.

**Dr. J. Apley** discussed the natural history of *The Infant with Stridor,* based on a follow-up survey of eighty cases. The importance of accurate diagnostic investigation was stressed, since the general term " congenital stridor " gives no information as to which of the many possible underlying causes is concerned. A familial tendency, mental retardation and neonatal upper respiratory infection were three factors in aetiology which had not been sufficiently recognized in the past. Complications were frequent, and the mortality rate was surprisingly high, in part because infants with stridor are traditionally and often wrongly, considered to be suffering from a trivial condition.

